# Vascular Ehlers-Danlos syndrome in children: evaluating the importance of diagnosis and follow-up during childhood

**DOI:** 10.1038/s41431-024-01773-x

**Published:** 2024-12-27

**Authors:** Niamh R. Wilkinson, Elena Cervi, Bart Wagner, Deborah Morris-Rosendahl, Duncan Baker, Harpaul Flora, Kate von Klemperer, Toby Andrew, Neeti Ghali, Fleur S. van Dijk

**Affiliations:** 1 National EDS Service, London North West University Healthcare NHS Trust, London, UK; 2 Department of Metabolism, Digestion and Reproduction, Section of Genetics and Genomics, Imperial College London, London, UK; 3 Centre for Inherited Cardiovascular Diseases, Great Ormond Street Hospital, London, UK; 4 Electron Microscopy Section, Histopathology Department, Royal Hallamshire Hospital, Sheffield Teaching Hospitals NHS Foundation Trust, London, UK; 5 Clinical Genetics and Genomics Laboratory, Royal Brompton Hospital, London, UK; 6 National Heart and Lung Institute, Imperial College London, London, UK; 7 Sheffield Diagnostic Genetics Service, Sheffield Children’s Hospital NHS Foundation Trust, Sheffield, UK; 8 Department of Vascular Surgery, Barts Heart Centre, London, UK; 9 Adult Congenital Heart Disease, Barts Heart Centre, London, UK

**Keywords:** Paediatrics, Genetic testing, Genetics research

## Abstract

Vascular Ehlers-Danlos syndrome (vEDS) is a rare inherited connective tissue disorder predominantly caused by pathogenic *COL3A1* variants. Characteristic arterial and intestinal fragility and generalised severe tissue friability can lead to clinical events from childhood. We highlight a paucity of literature regarding children diagnosed with vEDS, possibly explained by a restraint in predictive testing, and present data on 63 individuals (23 index cases) with a clinical and genetic diagnosis of vEDS in childhood (<18 years) to address this. Patients were identified through the National Ehlers-Danlos Syndrome (EDS) Service London. We report on 18 events in childhood, recorded in 13 individuals. First events occurred at a median age of 11 years (IQR 0–13) and genetic testing was initiated as a direct result of the first event in 11/13 cases. In the cohort majority, diagnosis was the result of familial genetic testing (55%). Our findings emphasise the importance of offering genetic testing in childhood when there is a positive family history of vEDS and/or features suggestive of a potential inherited connective tissue disorder. Diagnosis in childhood allows for follow-up surveillance and informed multi-disciplinary management, in addition to genetic counselling and patient-led management including lifestyle modification. As seen in adult cohorts, we anticipate children with vEDS will experience the same protective benefit afforded by early diagnosis and present preliminary data on follow-up in childhood. Formal evaluation of the impact that diagnosis of vEDS in childhood has on disease management is needed when sufficient data is internationally available.

## Introduction

Vascular Ehlers-Danlos syndrome (vEDS, OMIM #130050) is a rare autosomal dominant connective tissue disorder characterised by arterial and intestinal fragility and severe tissue friability, with an estimated prevalence of 1:90,000. Internationally recognised diagnostic criteria guide clinical diagnosis of vEDS, with major criteria outlined as (i) molecularly confirmed family history, (ii) arterial rupture at a young age, (iii) spontaneous bowel perforation, (iv) uterine rupture, (v) carotid-cavernous sinus fistula (CCSF). [1] A combination of major and/or minor criteria is suggestive of vEDS, however their presence can be variable. Clinical assessment, including family history, and subsequent genetic testing are essential in establishing a molecularly confirmed diagnosis, ensuring appropriate management, surveillance, and access to predictive testing in family members.

Pathogenic *COL3A1* gene variants cause vEDS, and in rare cases specific arginine-to-cysteine substitution variants in *COL1A1*. [2] *COL3A1* encodes the α1 chain of (pro)collagen type III (COLIII), an extracellular matrix (ECM) component expressed in the tissues of the dermis, vasculature, and that of multiple major organs. [3] COLIII is homotrimeric, consisting of three identical α1 chains which intertwine to form a triple helix. In vEDS, pathogenic glycine missense substitutions and splice-site variants leading to in-frame exon skipping are most frequently observed. [4, 5] The dominant negative mechanism of these variants disrupts the stable assembly of COLIII through interference of mutant protein with the wild-type protein, leading to degradation and decrease of wild-type collagen III by 87.5%. Alternatively, null variants, which induce premature termination and consequent nonsense-mediated decay (NMD), cause haploinsufficiency. In these instances, total COLIII protein production is reduced by 50%, as with whole gene deletions. Variant type and effect can influence phenotypic severity; null variants are reported to have a milder disease course but are less frequently identified. [6] Alternatively, splice-site variants which predominantly cause in-frame exon skipping and affect splice donor sites, are associated with a more severe phenotype. [4, 5] In glycine substitutions this is further influenced by properties of the amino acid that replaces the wild-type residue. [3, 5]

Frequently, life-threatening events in people with vEDS do not occur until adulthood where they are often the trigger for genetic investigations. Conversely, most vEDS diagnoses made in childhood are initiated due to positive family history. Little, however, has been reported on additional clinical characteristics in this young population, especially in those where diagnosis is not the result of familial testing or because of an event. Here, we aim to identify critical drivers of vEDS diagnosis in children by providing a detailed clinical and molecular description of 63 individuals with a molecularly confirmed diagnosis of vEDS in childhood, and highlight current management and surveillance strategies.

## Materials and methods

### Patients

Patients with a clinical and molecularly confirmed diagnosis of vEDS were identified through the National EDS Service in London, a quaternary service covering the South of England and Wales, which functions as an expert centre for diagnosis and follow-up of monogenic EDS types. Cohort data was obtained from the Natural history Exploration of rare EDS types (NEEDS) study and retrospective review of clinical activity in the London EDS service. Individuals with a (likely) pathogenic *COL3A1* variant diagnosed under the age of 18 years were eligible for the cohort study, reported herein.

Childhood cardiovascular surveillance is provided by Great Ormond Street Hospital (GOSH) in 48 individuals. Of those not seen at GOSH, *n* = 9 had no cardiology review in childhood (all now current adults under follow-up), *n* = 1 has yet to be seen, and *n* = 5 are followed -up by their local cardiology service. Current GOSH surveillance involves annual cardiology appointments, annual ECG and ECHO, and biennial arterial tree surveillance (head to knee MRA) starting at age 7–8 years in asymptomatic children, when able to tolerate without general anaesthetic, increasing to annual surveillance if symptomatic (Fig. S1). Blood pressure lowering medication with the beta-blocker celiprolol is offered as tolerated with recommendation to start at age 7–8 years, but ultimately decided on a case-by-case basis, considering the age of diagnosis, cardiovascular history, and parent/child wishes. When on treatment, an exercise test and 24-hour blood pressure monitoring every 1–2 years is conducted.

### Classification of clinical characteristics

Clinical characteristics were classified as major or minor as previously reported. [1] An event was defined as a symptomatic clinical event that required specialist management or intervention, that occurred spontaneously or would not have occurred to the same degree in an unaffected individual. All events reported are correct as of the last follow-up appointment. Clinical characteristics consisted of the number, type, and age of events, and the minor diagnostic criteria present on both physical examination and as reported by the patient/parents. Age of last follow up in childhood was used instead of current age as *n* = 24 individuals diagnosed in childhood are now adults. Information on events in current adults will be reported in a future adult cohort. Age of diagnosis was defined as age at confirmation by genetic testing. Presence of other remarkable features and medical histories, including minor surgical interventions and congenital anomalies, were also recorded.

### Genetic analysis

DNA analysis was completed prior to study commencement, between 1989 and 2023. Genetic data was available for all 63 individuals following DNA analysis of *COL3A1* by either Sanger sequencing and gene dosage analysis using multiplex ligation dependent probe amplification (MLPA, MRC-Holland) or next generation sequencing (NGS, NextSeq550 or Illumina HiSeq). Variants were classified according to ACMG criteria. For all relatives, targeted sequencing for the familial variant was performed. Variants were described at the nucleotide and protein levels, according to Human genome Variation Society nomenclature (HGVS, https://varnomen.hgvs.org/) and based on *COL3A1* gene sequence (NM_000090.4). Exons were defined following legacy nomenclature (exons 1–52) as used by the LOVD *COL3A1* database (https://databases.lovd.nl/shared/variants/COL3A1).

For analysis of genotype-phenotype associations, variants were categorised into six distinct subgroups based on variant type: [1] glycine substitutions in the triple helical domain; [2] splice-site variants; [3] non-glycine missense substitutions; [4] in-frame insertions, deletions, and duplications; [5] variants resulting in haploinsufficiency; [6] nonsense substitutions not resulting in haploinsufficiency. Variants resulting in haploinsufficiency were either frameshift deletion-insertions located in the C-terminal domain or interstitial deletions of >100 kilobases encompassing *COL3A1* and *COL5A2*. Group 6 variants were exclusively located in the C-terminal domain, within the terminal exon, and therefore escaped NMD and did not cause haploinsufficiency.

### Statistical analysis

Descriptive statistics and non-parametric tests were used to report quantitative variables (mean, median, interquartile range). Statistical analyses were performed using Microsoft Excel built-in statistics tools and R software (version 4.3.3), and a threshold of 0.05 was used to indicate nominal significance. In groups where less than 10% of data was unknown (i.e. classified as ‘not stated’), statistical analysis was performed using percentages reported as a function of total numbers.

## Results

A total of 63 individuals (23 index, 40 relatives) with vEDS who received molecular confirmation of a pathogenic *COL3A1* variant in childhood (<18 years) were studied (Table 1). This cohort included eight pairs of siblings and two trios of related individuals. Almost all individuals were of European origin, predominantly white (*n* = 38, 60.3%), with a similar proportion of males and females (33 M:30 F). The median age at last follow-up in childhood was 11 years (interquartile range [IQR] 7–16 years, range 1–18). Median age at molecular diagnosis was 7 years (IQR 2–13, range 0–17) and did not differ significantly between index cases and relatives. We report no deaths in childhood in this cohort. However, in addition to the 23 study index cases, we are also aware of one male with a post-mortem molecular diagnosis of vEDS following a lethal aortic rupture at age 14, whose family was referred to the London EDS service.

**Table 1:** Characteristics and complications of individuals with a clinical and molecular diagnosis of vEDS in childhood.

	All patients	Female	Male	F *vs* M	Index	Relatives	IC *vs* R
	*n* = 63	*n* = 30	*n* = 33	*P*-value	*n* = 23	*n* = 40	*P*-value
Median age at last follow up in childhood	11(7–16)	12 (6–15)	11 (8–17	–	9 (6–17)	13 (8–16)	–
Median age at molecular diagnosis	7 (2–13)	6 (2–13)	7 (3–12)	ns	7 (2-13)	7 (3–13)	ns
≥1 event in childhood	13 (21%)	4 (13%)	9 (27%)	ns	8 (35%)	5 (13%)	0.05
Median age at first event	11 (0–13)	6 (0–12)	11 (0–15)	ns	13 (8–14)	0 (0–9)	ns
≥1 vascular event in childhood	2 (3%)	1 (3%)	1 (3%)	ns	2 (9%)	0 (0%)	ns
≥1 gastrointestinal event in childhood	6 (10%)	1 (3%)	5 (15%)	ns	4 (17%)	2 (5%)	ns
≥1 other organ rupture in childhood^a^	6 (10%)	2 (7%)	4 (12%)	ns	3 (13%)	3 (8%)	ns

Qualitative variables displayed as medians (interquartile range). Median age given in years. Where supported, Wilcoxon tests (for age-dependent categories) and Fisher exact tests were used to analyse data, and the threshold for nominal significance was 0.05.

*F* female, *M* male. *IC* index case, *R* Relative.

^a^Other organ rupture events regarded spontaneous pneumothoraxes in *n* = 5 and splenic rupture in *n* = 1.

### Events in childhood

A total of 18 events were recorded in 13 individuals (Table 2). Single events were recorded in 9/13 (69.2%), and the maximum number of events for a single individual was three. Events occurred between 0–17 years. Events in the neonatal period were recorded in 4 individuals (*n* = 2 gastrointestinal perforation, *n* = 2 spontaneous pneumothorax), all of whom were born prematurely (median gestational age 35.3 weeks).

**Table 2:** Events occuring under the age of 18 years in individuals with a diagnosis of vEDS in childhood.

Variant group	Sex	IC/R	Event type	Event description	Age at event (years)	Event order	Post-operative complications	Premature birth
Group 1	F	IC	O	Splenic rupture	12	First event	+	−
Group 1	F	R	O	Spontaneous pneumothorax	2 days	First event	−	+
Group 1	M	IC	V	Popliteal artery rupture	13	First event	−	−
Group 1	M	IC	G	Caecal perforation	3 days	First event	−	+
Group 1	M	R	G	Gastric perforation	Neonatal period	First event	−	+
Group 1	M	R	G	Colonic perforation	9	First event	−	+
Group 1	M	IC	G	Sigmoid volvulus with ischaemia	11	First event	+	−
Group 1	M	IC	G	Adhesive bowel obstruction	15	Subsequent event	+	−
Group 1	M	R	O	Recurrent bilateral pneumothoraxes	2 days	First event	−	+
Group 2	M	IC	G	Small bowel obstruction	15	First event	+	U
Group 2	M	IC	G	Adhesive small bowel obstruction	15	Subsequent event	−	U
Group 2	M	IC	O	Spontaneous pneumothorax	17	Subsequent event	−	U
Group 2	M	IC	O	Right sided spontaneous pneumothorax	16	First event	−	+
Group 2	F	IC	G	Colonic perforation	13	First event	+	−
Group 2	F	IC	G	Colonic perforation	14	Subsequent event	−	−
Group 4	F	IC	V	Bilateral subdural haematoma and retinal haemorrhage	3 months	First event	−	+
Group 4	M	R	O	Pneumothorax in the setting of a lung abscess	15	First event	−	U
Group 4	M	R	O	Haemopneumothorax	17	Subsequent event	−	U

Variant groups were defined as: (Group 1) Glycine substitutions in the triple helical domain; (Group 2) Splice-site variants; (Group 3) Non-glycine missense substitutions; (Group 4) In-frame insertions, deletions, and duplications; (Group 5) variants resulting in haploinsufficiency; (Group 6) Nonsense substitutions not resulting in haploinsufficiency. Premature birth was defined as birth before 37 weeks of pregnancy.

*F* female, *M* male. *IC* Index case, *R* Relative. Event type: *V* vascular, *G* gastrointestinal, *O* Other.

All events required surgical intervention or repair and while no events were lethal, post-operative complications were reported in 5 individuals. Median age at first event was 11 years (IQR 0–13). Events were observed more frequently in males versus females (27.3% *vs*. 13.3%), and in index cases versus relatives (34.8% *vs*. 12.5%) but these differences were not statistically significant (Table 1). Event nature varied between males and females with notable differences in proportion seen with gastrointestinal events (5 M:1 F) and spontaneous pneumothoraxes (4 M:1 F). Two or more events were recorded in childhood in 3 individuals, two index males and one index female, all of whom experienced subsequent gastrointestinal events.

Events were most frequently gastrointestinal (*n* = 9 events in 6 children) and occurred at a median age of 13 (IQR 9–15). These events were predominantly perforations, affecting the sigmoid colon, caecum, and stomach, or adhesive small bowel obstructions. Vascular events were reported in 2 individuals, both experiencing spontaneous medium-sized vessel ruptures. There were no reports of aortopathy in this cohort. Other organ ruptures were also recorded and regarded splenic rupture (in *n* = 1) and spontaneous pneumothoraxes (*n* = 6 in 5 children) which occurred as young as 1 day old.

### Minor diagnostic criteria and additional clinical features

One or more minor diagnostic criteria were found to be present in childhood in 93.3% of individuals (Table 3), with a median of 3 minor criteria present across the total cohort (IQR 2–4, range 0–9). All minor criteria were observed at least once, except for keratoconus which was not reported. Excessive bruising (82.3%) and thin translucent skin (76.4%) were frequently observed. Additional clinical features in this cohort are detailed in Table S1. A total of 17 congenital defects were reported in 14 individuals (22.2%). In addition to bilateral congenital hip dislocation (*n* = 1, 1.6%) and bilateral talipes (*n* = 7, 11.1%), these included amniotic band sequence (*n* = 2, 3.2%), pyloric stenosis (*n* = 1, 1.6%), Chiari malformation type I (*n* = 1, 1.6%), and congenital heart defects (*n* = 4, 6.3%).

**Table 3:** Minor diagnostic criteria (2017 international classification of the Ehlers-Danlos syndromes criteria).

Minor criteria	All patients	Female	Male	F *vs* M	Index	Relatives	IC *vs* R
	*n* = 63	*n* = 30	*n* = 33	*P*-value	*n* = 23	*n* = 40	*P*-value
≥1 minor criteria present	56 (93%)^a^	27 (96%)^a^	29 (91%)^a^	ns	21 (95%)	35 (92%)^a^	ns
Median number of minor criteria	3 (2–4)	3 (2–3)	3 (2–4)	ns	3 (2.5–4)	3 (2–3)	ns
Excessive bruising	51 (82%)^a^	26 (87%)	25 (78%)^a^	ns	19 (83%)	32 (82%)^a^	ns
Thin translucent skin	42 (76%)^a^	22 (79%)^a^	20 (74%)^a^	ns	18 (81%)^a^	24 (73%)^a^	ns
Characteristic facial appearance^b^	28 (57%)^a^	13 (57%)^a^	15 (58%)^a^	ns	14 (70%)^a^	14 (48%)^a^	ns
Spontaneous pneumothorax	5 (8%)	1 (3%)	4 (12%)	ns	2 (8%)	3 (7%)	ns
Acrogeria	3 (5%)	1 (3%)	2 (6%)	ns	2 (8%)	1 (2%)	ns
Talipes equinovarus	7 (11%)	5 (17%)	2 (6%)	ns	2 (8%)	5 (12%)	ns
Congenital hip dislocation	1 (2%)	1 (3%)	0 (0%)	ns	1 (4%)	0 (0%)	ns
Hypermobility of small joints	40 (87%)^a^	18 (82%)^a^	22 (92%)^a^	ns	17 (94%)^a^	23 (82%)^a^	ns
Tendon rupture	3 (5%)	1 (3%)	2 (6%)	ns	0 (0%)	3 (8%)	ns
Gingival recession and fragility	9 (27%)^a^	6 (35%)^a^	3 (19%)^a^	ns	4 (27%)^a^	5 (28%)^a^	ns
Early onset varicose veins	1 (2%)	0 (0%)	1 (3%)	ns	1 (4%)	0 (0%)	ns

Percentages were calculated from available data.

*F* female, *M* male. *IC* index case, *R* relative.

^a^Denotes percentages calculated from known data, excluding individuals for whom there were no/insufficient comment on presence of specific minor diagnostic criteria.

^b^Characteristic facial appearance was determined by presence of prominent eyes and/or a combination of narrow nose, thin vermilion of the lips, micrognathia and tethered earlobes. For those where specific facial features were described, *n* = 26 (53.1%) had prominent/deep-set eyes, *n* = 7 (14.3%) had a narrow nose, *n* = 2 (4.1%) had thin lips, and *n* = 14 (28.6%) had tethered earlobes.

### Birth and early development

Information on birth and early development was available for 55 individuals. Premature birth occurred in *n* = 31 (55.4%), *n* = 18 (58.1%) of whom had an affected mother, and in *n* = 7 (12.7%, *n* = 6 females) preterm premature rupture of membranes (PPROM) was reported. Median gestational age was 36.7 weeks (IQR 35.3–39.0). Post-natal special care admissions, not related to an event, were required by 10 individuals (18.2%), all of whom were born prematurely. Description of a developmental delay was noted in *n* = 10 (19.6%). This involved delayed motor development in *n* = 4 (3/4 had foot deformities), delayed speech and language in *n* = 3, and global developmental delay in *n* = 3. All cases of global developmental delay were reported in individuals with traumatic premature births.

### Diagnosis of vEDS in childhood

In this cohort, we identified three pathways to a diagnosis of vEDS in childhood: (i) following an event, (ii) referral for familial testing, or (iii) investigation of specific clinical features. Clinical investigations and subsequent genetic testing were initiated as a direct result of the first event in 11/13 cases, *n* = 4 of whom had at least one 1st-degree relative diagnosed with vEDS at the time of the event. In most individuals (*n* = 35, 55.5%) diagnostic investigations were due to a positive family history of vEDS, initiated as part of familial genetic testing. Median age of diagnosis was 7 (IQR 3–13) and ≥1 minor diagnostic features were present in 90.9%.

In those individuals without a family history of vEDS or an event (*n* = 15, 23.8%), diagnosis occurred at a median age of 6 years (IQR 2–8). Referral to the London EDS service was predominantly the result of easy and/or excessive bruising (*n* = 9, 60%). This was present in addition to ≥1 clinical features and/or familial clinical history suggestive of a diagnosis of vEDS or another inherited connective tissue condition with overlapping features in 4 individuals (Table S2). Molecular diagnosis of vEDS was the result of an incidental finding in *n* = 2, and investigations were initiated due to family history of a vascular event in one individual. Three individuals were also initially investigated for suspicion of non-accidental injury (NAI), in association with excessive bruising (*n* = 2) or bleeding (*n* = 1), before a diagnosis of vEDS was established. Following clinical assessment by the London EDS service, one or more minor diagnostic criteria were identified in 13/15 individuals (Table S3).

### COL3A1 variant classification

**Fig. 1:**
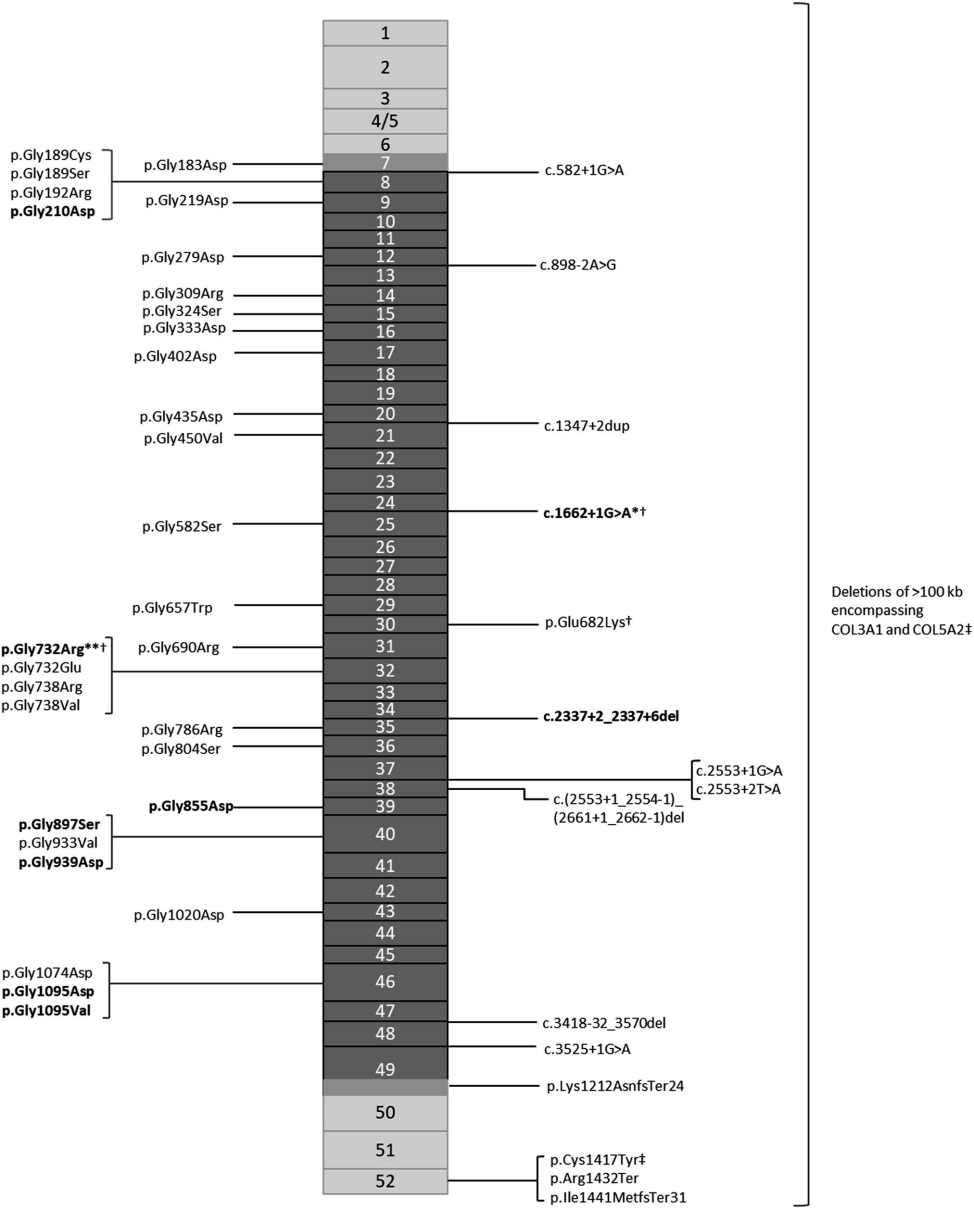
Schematic visualisation of 47 unique *COL3A1* (likely) pathogenic variants in 51 unrelated individuals. Boxes represent *COL3A1* exons, colour coded to define protein domains (N- and C-termini light blue; triple helix domain dark blue; transitional domains purple). Confirmed de novo variants are shown in bold. *de novo variant in 1/3 observed cases. **de novo variant in 2/2 observed cases. †denotes variants present in more than one unrelated individual. ‡ *n* = 2 deletions in unrelated cases: NC_000002.11:g.(?_187315204)_(192885646_?)del and NC_000002.11:g.(?_189828672)_(189931701_?)del.

The underlying genetic causes in all 63 individuals were likely pathogenic (*n* = 20) or pathogenic (*n* = 43) variants in the *COL3A1* gene (Table 4 and Table S4). 47 unique variants were identified, of which 3 were reported two or more times in index cases (Fig. 1). Variants spanned intron and exon regions across exons 7–52, with 84.1% of all variants located in regions encoding the triple helical domain. 77.8% (*n* = 51) of cases were inherited, *n* = 33 of maternal origin, and 12 variants were confirmed to be de novo.

**Table 4:** Clinical features and complications of individuals as categorised by type of *COL3A1* variant.

	*Group 1*. Gly missense substitutions	*Group 2*. splice variants	*Group 3*. non-Gly missense substitutions	*Group 4*. Ins/Del/Dup	Group 5. haploinsufficiency^b^	*Group 6*. nonsense substitutions
	*n* = 39	*n* = 10	*n* = 3	*n* = 2	*n* = 6	*n* = 3
Located in triple helix domain	39 (100%)	10 (100%)	2 (67%)	2 (100%)	0 (0%)	0 (0%)
Located in C-terminal domain	0 (0%)	0 (0%)	1 (33%)	0 (0%)	4 (67%)^a^	3 (100%)
Female sex	18 (46%)	5 (50%)	2 (67%)	1 (50%)	3 (50%)	1 (33%)
Index case	13 (33%)	8 (80%)	1 (33%)	1 (50%)	2 (33%)	0 (0%)
Confirmed de novo cases	9 (23%)	3 (30%)	1 (37%)	0 (0%)	0 (0%)	0 (0%)
Confirmed inherited cases	30 (77%)	6 (60%)	2 (100%)	2 (100%)	6 (100%)	3 (100%)
Median age at last follow up	11 (7–15)	16 (7–17)	8 (5–11)	12 (9–15)	16 (10–18)	14 (13–14)
Median age at diagnosis	7 (2–12)	8 (6–13)	4 (3–9)	8 (5–12)	10 (6–16)	11 (9–12)
≥1 event in childhood	8 (20%)	3 (30%)	0 (0%)	2 (100%)	0 (0%)	0 (0%)
Median age at first event	5 (0–11)	15 (14-16)	n/a	8 (4–11)	n/a	n/a
Vascular event	1 (5%)	0 (0%)	0 (0%)	1 (50%)	0 (0%)	0 (0%)
Gastrointestinal event	4 (11%)	2 (20%)	0 (0%)	0 (0%)	0 (0%)	0 (0%)
Organ rupture event	3 (7%)	2 (20%)	0 (0%)	1 (50%)	0 (0%)	0 (0%)
≥1 minor criteria present	33 (89%)	10 (100%)	3 (100%)	2 (100%)	4 (67%)	3 (100%)
Excessive bruising	30 (79%)^a^	9 (90%)	3 (100%)	2 (100%)	4 (67%)	3 (100%)
Thin translucent skin	27 (75%)^a^	8 (80%)	2 (67%)	2 (100%)	3 (75%)^a^	0 (0%)^a^
Characteristic facial appearance	16 (52%)^a^	7 (70%)	2 (67%)	1 (100%)^a^	2 (50%)^a^	0 (0%)^a^
Small joint hypermobility	24 (86%)^a^	9 (100%)^a^	2 (100%)^a^	2 (100%)^a^	1 (33%)^a^	2 (100%)^a^

Median age given in years. Percentages were calculated from available data.

^a^Denotes percentages calculated from known data, excluding individuals for whom there was no/insufficient comment on presence of specific minor diagnostic criteria.

^b^Variants resulting in haploinsufficiency were either frameshift deletion-insertions located in the C-terminal domain (*n* = 4), or interstitial deletions of >100 kilobases encompassing *COL3A1* and *COL5A2* (*n* = 2).

### Management of vEDS in children

53 individuals were recorded to have had one or more cardiology reviews in childhood, 79.2% of whom were described as asymptomatic from a cardiac perspective with no structural cardiac abnormalities identified. The median follow-up time in this cohort was 7.59 years, and no individuals were lost to follow up. New cardiovascular abnormalities were identified in 7 individuals while under surveillance and involved an aneurysm of the proximal superior mesenteric artery (*n* = 1), mitral valve prolapse (*n* = 1), and new dilations affecting either the ascending aorta (*n* = 2), aortic root (*n* = 1), left ventricle (*n* = 1), or jugular veins (*n* = 2) (Table S5). In individuals asymptomatic at point of baseline review (51/53, median age 10.2 years, IQR 4.13–14.33), the youngest age of identification of a new cardiovascular abnormality age 6 years (dilatation of internal jugular veins) followed by 8 years (dilatation of ascending aorta). 36 individuals are currently aged <18 years and all are under cardiac follow-up.

48 individuals were reviewed under GOSH surveillance (see methods and materials). A total of 34 individuals (70.8%) received blood pressure lowering medication in childhood. Individuals were primarily treated with celiprolol (*n* = 30) however other beta-blockers were also used (Atenolol = 2, Bisoprolol = 1, Propanolol = 1). In one individual angiotensin receptor blocker losartan was prescribed in combination with celiprolol to address systemic hypertension. Treatment was started at a median age of 12.53 years (IQR 10.53–15.10). 14 individuals were not on medication; treatment had not yet started due to age in *n* = 12 (median age 6.83 years), was not tolerated in *n* = 1, and was declined in *n* = 1. All individuals who experienced an event in childhood are currently taking blood pressure lowering medication.

## Discussion

We describe the first clinically driven paediatric series of individuals with a molecularly confirmed diagnosis of vEDS in childhood, a population which has yet to be extensively described.

### Events

Through description of events in 13 individuals (20.6%) we show that events, though infrequent, can occur in childhood. These findings are comparable to previous reports of event frequency in childhood, in which events had occurred in 12–24% of affected individuals by the age of 20 years. [4, 5] Furthermore, we observed that events can happen as early as the neonatal period, an age at which few events have been previously reported. [7] These could have occurred secondary to prematurity, especially in cases where invasive postnatal interventions such as ventilation are required as these could increase the risk of an event. To our knowledge there have been no published vEDS cohorts which report on events in childhood in the context of premature birth, however Stephens et al. [8] reports on increased rates of prematurity in individuals with vEDS, irrespective of maternal affected status.

Within this cohort, first events occurred at either ≤3 months (*n* = 5) or aged 9–16 years (*n* = 8), with no clear segregation in event type observed between these two groups. Total events were predominantly gastrointestinal (47.1%) or spontaneous pneumothoraxes (35.3%) and were observed more frequently in males, including three males who experienced ≥2 events in childhood. Vascular events were observed in only two individuals and did not include any aortic events, however arterial/aortic pathology in children with vEDS has been described. [5, 9]

This cohort likely provides a conservative estimate of event frequency in childhood (20.6%). This is because children in this cohort are of different ages. Therefore, although some individuals reported herein are now adults, the majority are still children (57%) and may still have events in childhood. Furthermore, individuals who have had lethal events before diagnosis of vEDS in life will not have been ascertained in these cohorts. This highlights the importance of genetic testing after death in individuals with complications suggestive of vEDS and related conditions and the implications this can have for future testing and management of biological family members. [10] In addition, at least 8 individuals diagnosed in childhood and managed by the London EDS service have experienced events between the ages of 18–25 years, *n* = 4 of whom have had lethal vascular events. Findings from some previous cohorts point to an increased risk of early death due to vascular events in young males. [5, 11, 12] Studies into cellular signalling in vEDS mouse models suggested a possible hormonal mechanism, with data showing a tentative relationship between increased risk of arterial rupture and puberty. [13] To further investigate the role sex may have in event type, age at event, and lethality, it is essential that children with vEDS are included in future cohort studies and details of events in childhood are reported.

### Minor diagnostic criteria

Easy and/or excessive bruising and thin translucent skin were the most common minor diagnostic criteria present and were observed in most individuals, as seen in previous cohorts. [4, 5] Characteristic facial features, determined by presence of a combination of prominent eyes, narrow nose, thin vermilion of the lips, and micrognathia, were observed in greater than 50% of the cohort, however this is less frequent than expected. [4, 14] Facial features were markedly varied between individuals and not always present or distinguishable at time of diagnosis. Similarly, we observed variability in joint hypermobility; while small joint hypermobility was present in the majority of this young population, we noted that in some individuals this was limited to only very small joints (such as metacarpophalangeal joint) whereas in others it was much more generalised. These findings show that vEDS minor criteria are variable in both presence and nature and, because paediatric vEDS cohorts have not been so extensively described, diagnostic criteria are potentially more aimed at adults with vEDS than children. This should therefore be taken into consideration when clinical and diagnostic investigations are undertaken.

In this cohort we identified additional clinical features which were over-represented versus general population frequency. Alongside talipes and congenital hip dislocation, further congenital abnormalities included the following congenital heart defects (CHDs): atrial septal defect (ASD), patent ductus arteriosus (PDA), pulmonary atresia with intact ventricular septum (PA/IVS), and ventricular septal defect (VSD). CHDs have previously been reported in neonates with vEDS [8] however the relationship between CHDs and *COL3A1* variants is not well understood and requires further investigation to determine whether an association can be made between CHDs and vEDS. Additional features in this cohort included amniotic band sequence (ABS) in 2 individuals. This feature has been previously reported in vEDS [15, 16] with a potential mechanism of collagen III reduction caused by pathogenic *COL3A1* variants leading to decreased structural integrity of the foetal-derived amnion. [8] Where available (55/63 individuals), characteristics of birth and early development were also interrogated. Features in neonates with vEDS have been detailed in prior publications [8, 17] where an over-representation of preterm birth was reported. In this cohort, premature birth was known to have occurred in 55.4%, with spontaneous PPROM noted in 12.7%. These clinical features may therefore be suitable early indicators of vEDS, as prior findings by Bowen et al. [14] also suggest, and a diagnosis of vEDS should be considered in children when present alongside other minor diagnostic criteria.

### Genotype-phenotype associations

Missense glycine substitutions (Group 1) were the most frequent variant type (61.9%) and individuals with Group 1 variants accounted for 61.5% of events in childhood in this cohort. Interestingly in these individuals, glycine was always substituted for bulkier amino acids aspartic acid (*n* = 5), valine (*n* = 2), and arginine (*n* = 1), which have been shown to be more destabilising to the collagen triple helix than other amino acids known to be substituted for glycine in pathogenic *COL3A1* variants, [18] and have been associated with a more severe phenotype. [3, 5] In the remaining individuals who experienced an event in childhood, *COL3A1* variants were located within the triple helix and were Group 2 (splice-site) or Group 4 (in-frame ins/del/dup) variants. In individuals with variants resulting in haploinsufficiency (Group 5) we observed presence of fewer minor diagnostic criteria and no events which may support associations with a milder disease course in these individuals. [6] Similarly, we also identified 3 nonsense substitutions within the C-terminal domain whose location within the terminal exon facilitates escape of NMD and are therefore not causing loss-of function, in whom we report no events so far in childhood. Clinical and molecular characteristics were assessed across each of the six variant groups however robust intergroup comparisons could not be drawn as variable sub-group sizes and low numbers did not provide sufficient power for statistical analysis. This therefore emphasises the benefit of future meta-analysis of vEDS cohorts. Furthermore, since individuals ascertained for the study were diagnosed in childhood, many are still aged less than 18 years (57%). For this reason, certain characteristics of this population may be under-represented, including frequency of events. As such, it is important for children with a diagnosis of vEDS to be included in longitudinal studies to provide a representative picture of disease course, survival, and genotype-phenotype correlations which may emerge through this data.

### Drivers of vEDS diagnosis in childhood

Our data shows that diagnosis of vEDS in childhood is most likely to be initiated by positive family history, with familial genetic testing occurring in 55%. It is possible that this represents a restraint in predictive testing. Such restraint in testing has been suggested by others [19] and could contribute to lower total ascertainment of children with vEDS through predictive testing. We speculate that potential restraint may be shown by clinicians due to the potential psychological burden of this diagnosis for children and parents, the perception that complications of vEDS typically have their onset in adulthood, and lack of management guidelines for children with vEDS.

In individuals without a positive family history of vEDS or an event, we identified that easy and/or excessive bruising was the most common initiator for referral for diagnostic investigations. This appears in line with observations in a cohort of 295 children with vEDS described by Pepin et al. [5] in whom easy bruising, thin skin, joint hypermobility, and talipes were the most frequent minor criteria to initiate diagnostic investigation in the absence of a positive family history or an event. First interactions with clinicians in these cases were often for haematological investigations, including testing for platelet disorders, and subsequent referral to clinical genetics teams and/or the London EDS service upon identification of other clinical features indicative of a potential inherited connective tissue disorder. A diagnosis of vEDS may therefore be considered in children with excessive bruising but normal haematological investigation, especially when one other minor diagnostic criterium of vEDS is also present. As such, *COL3A1* could be added to gene panels incorporating genetic causes of bleeding and platelet disorders. This cohort also details instances of NAI investigations due to severe bruising. It is therefore important to consider that there may be an underlying rare genetic cause in instances of NAI and taking family history is therefore crucial in these instances as a parent could exhibit similar symptoms and be affected with vEDS without knowing.

### Management of vEDS in children

Following diagnosis of vEDS in childhood by the London EDS service, individuals are primarily referred to the Inherited Cardiovascular Disease Clinic at GOSH where cardiovascular management is discussed, and individuals are followed-up in joint vEDS clinics with the London EDS service team. The proposed treatment and surveillance is mainly a consensus between authors, and is also based on recommendations in the literature. To date, no standardised international guidelines for management and surveillance in children with vEDS have been published, however specialists involved in their clinical care recommend periodic surveillance, by scanning of the aorta and arterial tree, and some form of blood pressure lowering medication. [20, 21]

Management of vEDS also extends beyond cardiovascular care. In this cohort a frequent gastrointestinal symptom in childhood was constipation and subsequent perirectal bleeding. To reduce the risk of gastrointestinal events, advice regarding diet and use of laxative medications to prevent constipation often form an essential part of condition management in individuals with these symptoms. Joint hypermobility and joint pain can have significant impact on physical activity, especially in children. This can be addressed by rheumatology assessment, physiotherapy, and orthotics. Furthermore, involvement of haematology teams has proven to be beneficial in children who experience excessive bleeding, recurrent nosebleeds, or menorrhagia, and who can benefit from expert advice which may include access to tranexamic acid when needed.

The impact of early diagnosis and effective management of vEDS in children is felt particularly by parents, who play significant roles in implementing supportive measures for their child such as suggesting appropriate moderate aerobic exercise, being aware of signs and symptoms that require medical attention, and ensuring necessary information is provided to nursery/school. To help alleviate some of the psychological burden diagnosis of vEDS can place on children and their family, additional support is provided by the London EDS service in the form of genetic counselling, information resources for patients/parents to provide health care professionals in both routine and emergency settings, and signposting to patient charities. [22]

## Conclusion

We present a large clinically driven paediatric cohort reporting on features of individuals diagnosed with vEDS in childhood. The study confirms events, though less frequent than in the adult vEDS population, can occur in childhood as early as the neonatal period and can require significant surgical intervention. In the UK, testing for vEDS in the context of positive family history is possible, with appropriate counselling, through the National Health Service to parents regardless of their child’s age. Findings from this cohort emphasise the importance of genetic testing in childhood when there is a positive family history of vEDS and/or features suggestive of a potential inherited connective tissue disorder. Diagnosis in childhood allows for management and follow-up, in addition to counselling for both children and their family. This can have a positive impact on disease course through factors including: (i) awareness of vEDS diagnosis by health care professionals, (ii) lifestyle modification, and (iii) potential influence of medication and surveillance. Formal evaluation of the impact that diagnosis of vEDS in childhood has on disease management is needed when sufficient data is internationally available. We anticipate that this will show an effect on decreasing morbidity and mortality, as seen when adults with vEDS are diagnosed and offered structural disease management by an expert service. [14, 23]

## Supplementary information

Supplemental Material

## Data Availability

The datasets analysed during the current study are available from the corresponding author on reasonable request. Variant data is openly available in LOVD: *COL3A1* at https://databases.lovd.nl/shared/genes/COL3A1.
