# Supplementary material for: Vascular Ehlers-Danlos syndrome in children: evaluating the importance of diagnosis and follow-up during childhood
Source: Eur J Hum Genet. 2024 Dec 27;33(3):368–76. doi: 10.1038/s41431-024-01773-x (PMC11893879; doi:10.1038/s41431-024-01773-x)
Supplement: Supplementary file 1 — Supplemental Material [file 41431_2024_1773_MOESM1_ESM.pdf]

Figure S1. Summary of GOSH cardiovascular surveillance and treatment for children with a diagnosis of vEDS

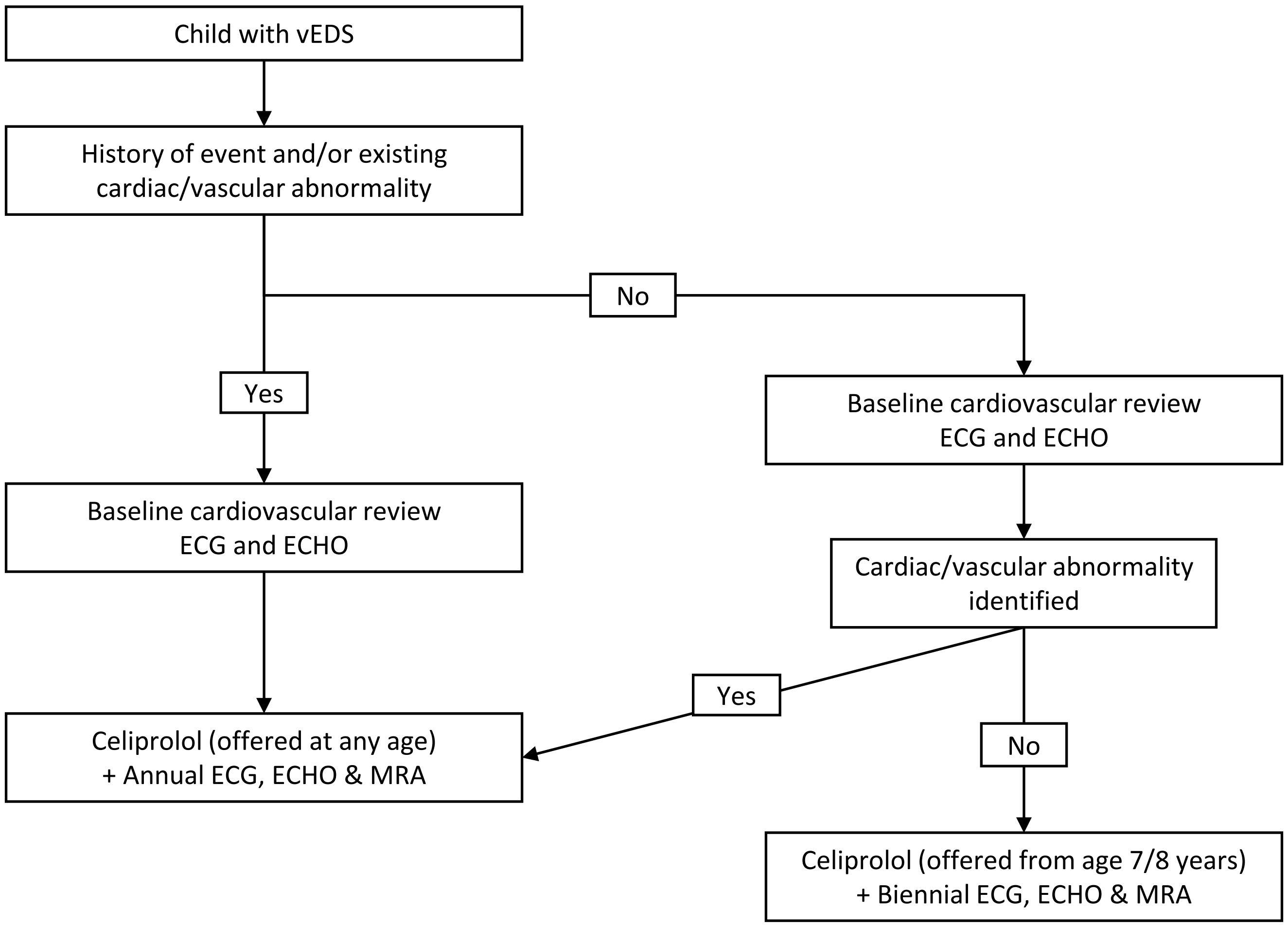

Table S1. Additional clinical features present in childhood in 63 individuals with a diagnosis of vEDS under the age of 18 years

Additional clinical features	All patients (n=63)
**Skin**	
Soft and/or hyperextensible skin	10 (16%)
Abnormal scarring	13 (21%)<br>Atrophic n=5<br>Keloid n=2<br>Hypertrophic n=1<br>Papyrous n=2<br>Undefined n=3
Hernia	7 (11%)<br>Inguinal n=3<br>Umbilical n=2<br>Hiatus n=1<br>Undefined n=1
**Connective tissue complications**	
Dislocation/subluxation	13 (21%)
Pes planus	27 (43%)
Joint pain	19 (30%)
**Congenital defects**	
Amniotic band sequence	2 (3%)
Pyloric stenosis	1 (2%)
Chiari malformation type I	1 (2%)
Congenital knee deformities	2 (3%)
Congenital heart defect	4 (6.3%)<br>Atrial septal defect (ASD) n=2<br>Patent ductus arteriosus (PDA) n=1<br>Pulmonary atresia with intact ventricular septum (PA-IVS) n=1<br>Ventricular septal defect (VSD) n=1

Table S2. Reasons for referral to the EDS service London in 15 individuals in whom there was no family history of vEDS or history of a major clinical event

	Bleeding	Bruising	Hypermobility	FH Vascular Event	FH Gastrointestinal event	Other skin features	Other clinical features	Incidental finding†	NAI
12	✓	✓							
18		✓							✓
19		✓	✓		✓				
22		✓	✓						✓
23			✓			✓ EDS specific			
25						✓ Connective tissue	✓ Connective tissue		
26		✓							
28		✓*							
30								✓	
31				✓					
37		✓					✓ Thin skin		
44		✓							
54		✓	✓						
57			✓			✓ EDS specific			
61								✓	

†Incidental findings resulting from genetic testing for clinical diagnoses not associated with connective tissue disorders and/or vEDS
*R90 Bleeding and platelet disorders genetic testing panel (https://panelapp.genomicsengland.co.uk/panels/545/)

Table S3. Minor diagnostic criteria (2017 international classification of the Ehlers–Danlos syndromes criteria) in 15 individuals in whom there was no family history of vEDS or history of a major clinical event

	Bruising	Thin skin	Facial features	Spontaneous pneumothorax	Acrogeria	Talipes	Congenital hip dislocation	Small joint hypermobility	Tendon rupture	Keratoconus	Gingival recession/ fragility	Early onset varicose veins
12	✓	✓	✓			✓		✓			U	
18	✓		✓			✓		✓			✓	
19	✓	✓						✓				
22	✓	✓	✓					✓				
23	✓	✓	✓					U			✓	
25								U			U	
26	✓	✓	U					✓			✓	
28	✓	✓	✓					✓				
30		✓						✓				
31	✓	✓	✓		✓		✓	U			U	
37	✓	✓	✓					✓				
44	✓	U	U					U			U	
54	✓	✓	✓					✓			✓	
57								✓			U	
61†												

Unknown data represented by 'U' and defines cases in which presence or absence of a feature was not ascertained. †Incidental finding in which familial testing identified an affected parent.

Table S4. Clinical characteristics of individuals with missense glycine substitutions based on the observed glycine-substituting residue

Order of destabilisation‡ →

	**Ser n=5**	**Cys n=2**	**Arg n=9**	**Val n=5**	**Glu n=2**	**Asp n=15**	**Trp n=1**
Female	3 (60%)	2 (100%)	2 (22%)	4 (80%)	1 (50%)	5 (33%)	1 (100%)
Index cases	2 (40%)	1 (50%)	2 (22%)	2 (40%)	0 (0%)	6 (40%)	0 (0%)
De novo	1 (20%)	0 (0%)	2 (22%)	1 (20%)	0 (0%)	5 (33%)	0 (0%)
Median age at last follow-up	17 (16-18)	2 (2-3)	7 (4-12)	13 (11-15)	10 (10-10)	14 (11-15)	[8]
Family history of vEDS†	4 (80%)	2 (100%)	7 (78%)	4 (80%)	2 (100%)	10 (71%)*	1 (100%)
Median age at diagnosis	14 (7-15)	1 (0.6-1)	3 (3-10)	10 (7-12)	0.1 (0.1-0.1)	7 (2-12)	[3]
≥1 major event in childhood	0 (0%)	0 (0%)	1 (11%)	2 (40%)	0 (0%)	5 (33%)	0 (0%)
Vascular event	0 (0%)	0 (0%)	0 (0%)	0 (0%)	0 (0%)	1 (20%)	0 (0%)
Gastrointestinal event	0 (0%)	0 (0%)	0 (0%)	1 (50%)	0 (0%)	3 (60%)	0 (0%)
Other event	0 (0%)	0 (0%)	1 (100%)	1 (50%)	0 (0%)	1 (20%)	0 (0%)

Median age given in years. ‡ Order of residue destabilisation of the COLIII triple helix from least (left) to most (right) as outlined by Persikov *et al.* (2004). †Family history of vEDS defined as individuals with at least one first-degree relative with a molecularly confirmed diagnosis of vEDS. *Family history unknown in n=1

Table S5. Summary of new cardiovascular abnormalities identified while under surveillance in childhood

Location	Cardiovascular abnormality	Measurement (mm)	Z-score	
			Kaiser	Lopez
Proximal superior mesenteric artery	Aneurysm	6		
Mitral valve	Prolapse	-	-	-
External and internal jugular veins‡	Dilation	-	-	-
Ascending aorta	Dilation		+4.4	
Left ventricle	Dilation		+2.3	
Internal jugular veins	Dilation	28 (R) 21 (L)		
Aortic root	Dilation		+2.16	
Proximal ascending aorta	Dilation			+3.2

‡Measurement/z-score unknown
